# The expression of presenilin 1 enhances carcinogenesis and metastasis in gastric cancer

**DOI:** 10.18632/oncotarget.7298

**Published:** 2016-02-10

**Authors:** Ping Li, Xi Lin, Jun-Rong Zhang, Yun Li, Jun Lu, Fei-Chao Huang, Chao-Hui Zheng, Jian-Wei Xie, Jia-Bin Wang, Chang-Ming Huang

**Affiliations:** ^1^ Department of Gastric Surgery, Fujian Medical University Union Hospital, Fuzhou, People's Republic of China; ^2^ Key Laboratory of the Ministry of Education for Gastrointestinal Cancer, School of Basic Medical Sciences, Fujian Medical University, Fuzhou, People's Republic of China

**Keywords:** PS-1, gastric cancer, tumorigenicity, E-cadherin, β-catenin

## Abstract

Presenilin 1 (PS-1, encoded by *PSEN1*) is a part of the gamma− (γ−) secretase complex. Mutations in *PSEN1* cause the majority of cases of familial Alzheimer's disease (FAD). Although in recent years PS-1 has been implicated as a tumor enhancer in various cancers, nothing is known regarding its role in gastric cancer (GC). In the present study, we investigate the role and clinical significance of PS-1 in GC. We observed that PS-1 was significantly upregulated and amplified in GC tissues and cell lines, and its aberrant expression was positively correlated with lymph node metastasis and with poor overall survival. Furthermore, PS-1 promoted tumor invasion and metastasis of GC both *in vitro* and *vivo* without affecting the proliferation of GC cells (MGC-803 and MKN-45). The results of treatment with the γ-secretase inhibitor DAPT were consistent with the outcomes of PS-1 silencing. PS-1/γ-secretase cleaves E-cadherin and releases its bound protein partner, β-catenin, from the actin cytoskeleton, thereby allowing it to translocate into the nucleus and to activate the TCF/LEF-1 transcriptional activator, which may promote GC invasion and metastasis.

In conclusion, PS-1 promotes invasion and metastasis in GC and may represent a novel prognostic biomarker and potential therapeutic target for GC treatment.

## INTRODUCTION

Presenilin 1 (PS-1, encoded by *PSEN1*), a ubiquitously expressed multi-transmembrane domain protein, is primarily located on the endoplasmic reticulum (ER), Golgi apparatus, and plasma membrane. *PSEN1* mutations account for the majority of early-onset familial Alzheimer's disease [1–3]. PS-1, distinct from nicastrin (NCT), anterior pharynx defective-1 (Aph-1), and presenilin enhancer 2 (PS-2), functions as a core catalytic subunit of the γ-secretase complex that is involved in the cleavage of several type-I transmembrane proteins, including β-amyloid precursor protein (APP), Notch, CD44, Vascular Endothelial Growth Factor Receptor (VEGFR), E-cadherin and N-cadherin [4–9]. With the cleavage of PS-1/γ-secretase, gradual accumulation of APP would lead to the progression of Alzheimer's disease. Recent studies have revealed multiple common pathways involved in Alzheimer's disease and cancer developments [10]. PS-1 plays an exclusive and significant role in various tumorigenic processes including cell proliferation, apoptosis, cell adhesion and others [11, 12]. Previous studies have revealed diverse, even controversial, functions of PS-1 in various cancers dependent or independent of γ-secretase activity. In head and neck squamous cell carcinoma, PS-1 positively modulates epidermal growth factor receptor (EGFR) expression independently of γ-secretase cleavage, whereas downregulation of PS-1 can inhibit the EGFR-STAT pathway [13]. Enhanced expression of proteolytically active PS-1 is associated with E-cadherin proteolysis and nuclear translocation, which promotes peritoneal metastasis in colorectal cancer [14]. However, conflicting results were obtained for breast and skin cancer [15, 16], in which PS-1 acted as a tumor suppressor. The tissue-specific micro-environments in which different cancers develop may explain the seemingly contradictory roles of PS-1. Nevertheless, for now, the role that PS-1 plays in GC remains unknown.

Gastric cancer (GC) is the second leading cause of cancer-related death worldwide, particularly in East Asia, with a high rate of incidence that ranges from 40 to 60 cases per 100,000 residents [17, 18]. The prognosis is poor, with an average 5-year survival rate of no more than 20%, mainly because of late-stage diagnosis and the lack of sensitive biomarkers for early detection. Herceptin has proven to be beneficial to GC patients with greater expression of EGFR and HER2 [19]. In the same way, γ-secretase inhibitors (GSIs) have been investigated as therapeutic agents in various cancers, including pancreatic ductal adenocarcinoma, T cell acute lymphoblastic leukemia, and non-small cell lung carcinoma [20–22]. The therapeutic activity of GSIs is partly attributed to an enhanced sensitivity to chemotherapy and inhibition of Notch signaling. DAPT, another type of γ secretase inhibitor, has also been used to prevent the tumorigenesis of GC cells by inhibiting the Notch signaling pathway and the epithelial-mesenchymal transition (EMT) [23]. As one of the hydrolysis substrates of the PS-1/γ-secretase complex, E-cadherin plays important roles in cell invasion, proliferation and differentiation [8]. E-cad/CTF-2 (E-cadherin C-terminal fragment-2), the product of full-length E-cadherin cleavage by PS-1, can bind to β-catenin [24]. Abnormal β-catenin expression also correlates with E-cadherin, and aberrations in both proteins have been observed in diffuse-histotype or poorly differentiated GC [21]. Nevertheless, no studies have examined the relationship between PS-1, E-cadherin and β-catenin in GC. In this study, we measure the expression of PS-1 in GC and in adjacent tissues. We demonstrate that PS-1 is a tumor enhancer in GC and affects cell invasion and migration but not cell proliferation. PS-1 may contribute to the tumorigenesis of GC in a γ-secretase-dependent manner by regulating E-cadherin cleavage and β-catenin nuclear accumulation, which plays a key signaling role in the activation of TCF/LEF-1.

## RESULTS

### Expression of PS-1 in GC tissues and cells

**Figure 1:**
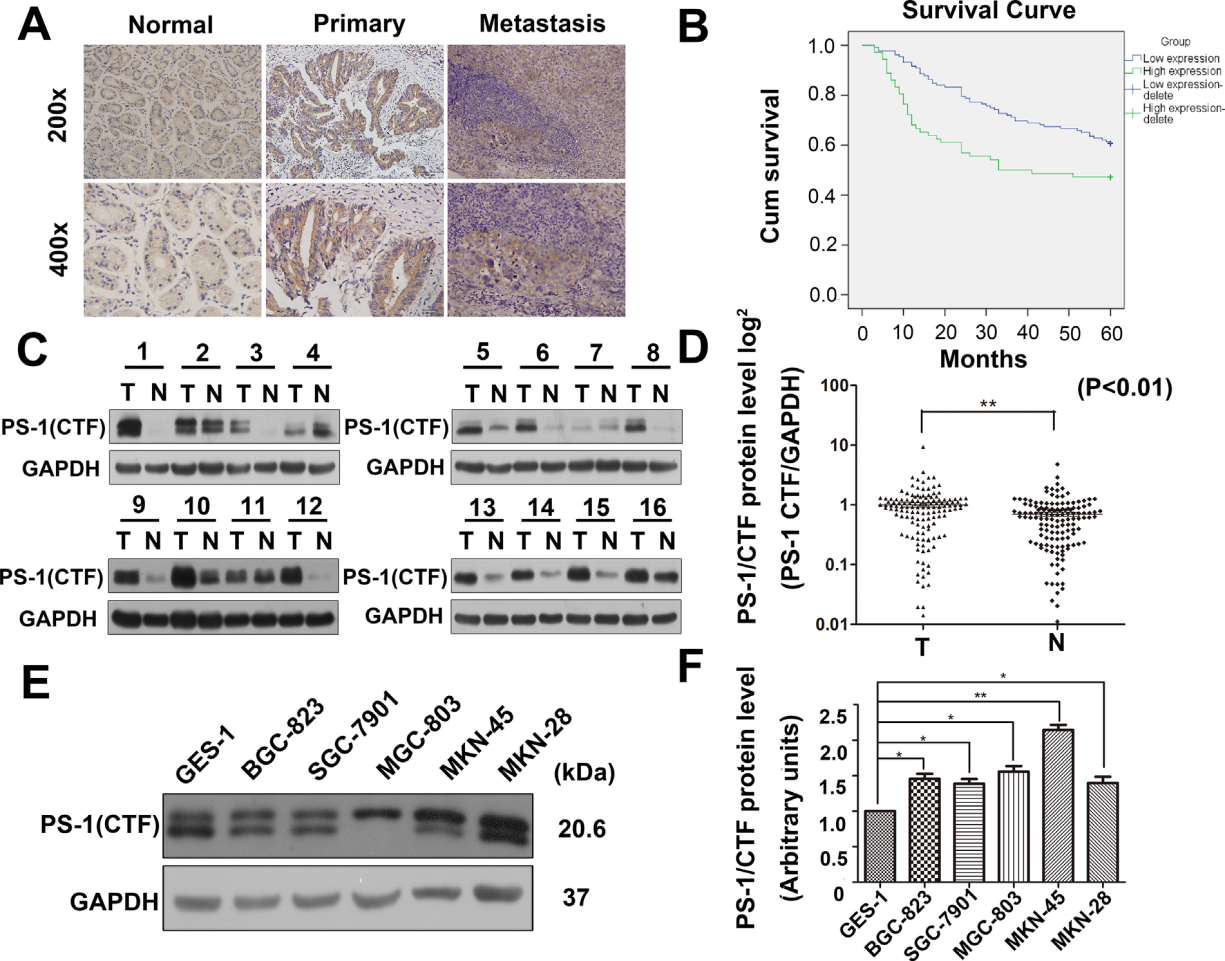
Upregulation of PS-1(CTF) correlates with poor prognosis in human GC (**A**) Representative PS-1(CTF) immunostaining in normal gastric tissues, primary gastric tumors, and lymph node metastasis. Picture magnification 200× (Top) and 400× (Bottom). (**B**) Patients with PS-1(CTF) strong staining have a significantly poorer prognosis than those with weak staining (**p < 0.01). (**C**) Western blot analysis of PS-1(CTF) expression in 136 pairs of gastric tumor (T) and adjacent non-tumor mucosa (N). Equal loading of proteins are determined by GAPDH. (**D**) PS-1(CTF) levels were quantified by densitometry. The expression of PS-1(CTF) in gastric tumors and paired non-tumor tissues from 136 patients are presented (**p < 0.01). (**E**) Expression of PS-1 in human GC cell lines and the normal gastric epithelial cell line(GES-1), the quantification of the expression is analyzed in (**F**).

To evaluate the prognostic role of PS-1 in human GC from our clinical data, we used immunohistochemistry (IHC) to examine 204 paraffin-embedded, archived GC tissue samples from patients who underwent surgery at least 5 years ago and who had paired, detailed pathologic scoring records. As shown in Figure 1A and Table 1, PS-1 was markedly upregulated in GC tissues and the relevant metastatic lymph nodes but marginally detectable in matched adjacent non-tumor mucosa (*p* < 0.01). Based on the expression of PS-1 in the GC tissue, we classified samples into 2 groups: higher PS-1 and lower PS-1. Of the 204 cases, 72 with increased expression of PS-1 and IHC scores greater than 4 points were defined as the “higher PS-1” group. The remaining 132 cases were classified into the “lower PS-1” group (with IHC scores no higher than 3 points). Five-year survival analysis showed that patients in the “high PS-1” group had a much shorter survival rate than the patients in the “lower PS-1” group (Figure 1B), indicating that PS-1 is a prognostic indicator for long-term survival outcome.

**Table 1:** Clinicopathological parameters of GC patients with different PS-1 expression

Clinicopathological Parameters	No. of patients	PS-1 expression		*X^2^*	*p*
		Lower	Higher		
**Normal vs cancer**				45.44	0.000*
Normal	204	188	16		
Cancer	204	132	72		
**Age (year)**					
Mean (SEM)		60.33 (0.840)	60.81 (1.340)	0.317	0.751
**Gender**				0.124	0.725
Male	150	96	54		
Female	54	36	18		
**Location**				2.117	0.548
Upper	52	35	17		
Middle	34	21	13		
Lower	103	64	39		
Mixed	15	12	3		
**Tumor size (cm)**				1.033	0.309
≤ 5	134	90	44		
> 5	70	42	28		
**Borrmann type**				1.387	0.239
I + II	82	57	25		
III + IV	122	75	47		
**Grade of differentiation**				1.699	0.192
Well and moderate	115	70	45		
Poor and not	89	62	27		
**Depth of invasion**				4.081	0.253
T1	41	29	12		
T2	36	24	12		
T3	9	8	1		
T4	118	71	47		
**pTNM stage**				6.899	0.066
I	62	44	18		
II	27	21	6		
III	111	66	45		
IV	4	1	3		
**Lymphatic metastasis**				14.672	0.002**
N0	77	60	17		
N1	25	17	8		
N2	33	22	11		
N3	69	33	36		

** *P* < 0.01, statistical significance.

To confirm the data from IHC, we also examined the expression of PS-1 in fresh human GC tissue samples and cell lines via Western blotting. After analysis of an additional 136 consecutive surgical biopsies, we observed that GC tissues exhibited significantly higher amounts of PS-1 expression compared with adjacent non-tumor mucosa (89 of 136, 65.7%, *p* < 0.01) (Figure 1C and 1D), consistent with the IHC results. In addition, to confirm these findings in GC cell lines, we investigated PS-1 expression in five GC cell lines (BGC-803, SGC-7901, MGC-803, MKN-45 and MKN-48) and one normal gastric mucosal epithelial cell line (GES-1). In comparison to GES-1, the expression of PS-1 was upregulated in the GC cell lines, particularly in MGC-803 (*p* < 0.05) and MKN-45 (*p* < 0.01) (Figure 1E and 1F).

We also investigated the association between PS-1 expression and clinic-pathological characteristics (Table 1). High expression of PS-1 in GC exclusively correlated with lymph node involvement (*p* < 0.002), consistent with our previous findings on survival. However, no statistically significant relationships between PS-1 expression and other clinicopathological variables, such as tumor size, histological grade and TNM stage, were observed. Taken together, these findings indicate that PS-1 is highly expressed in GC tissues and cell lines and suggests a potential link between PS-1 overexpression and GC progression.

### Alteration of PS-1 does not affect GC cell proliferation

**Figure 2:**
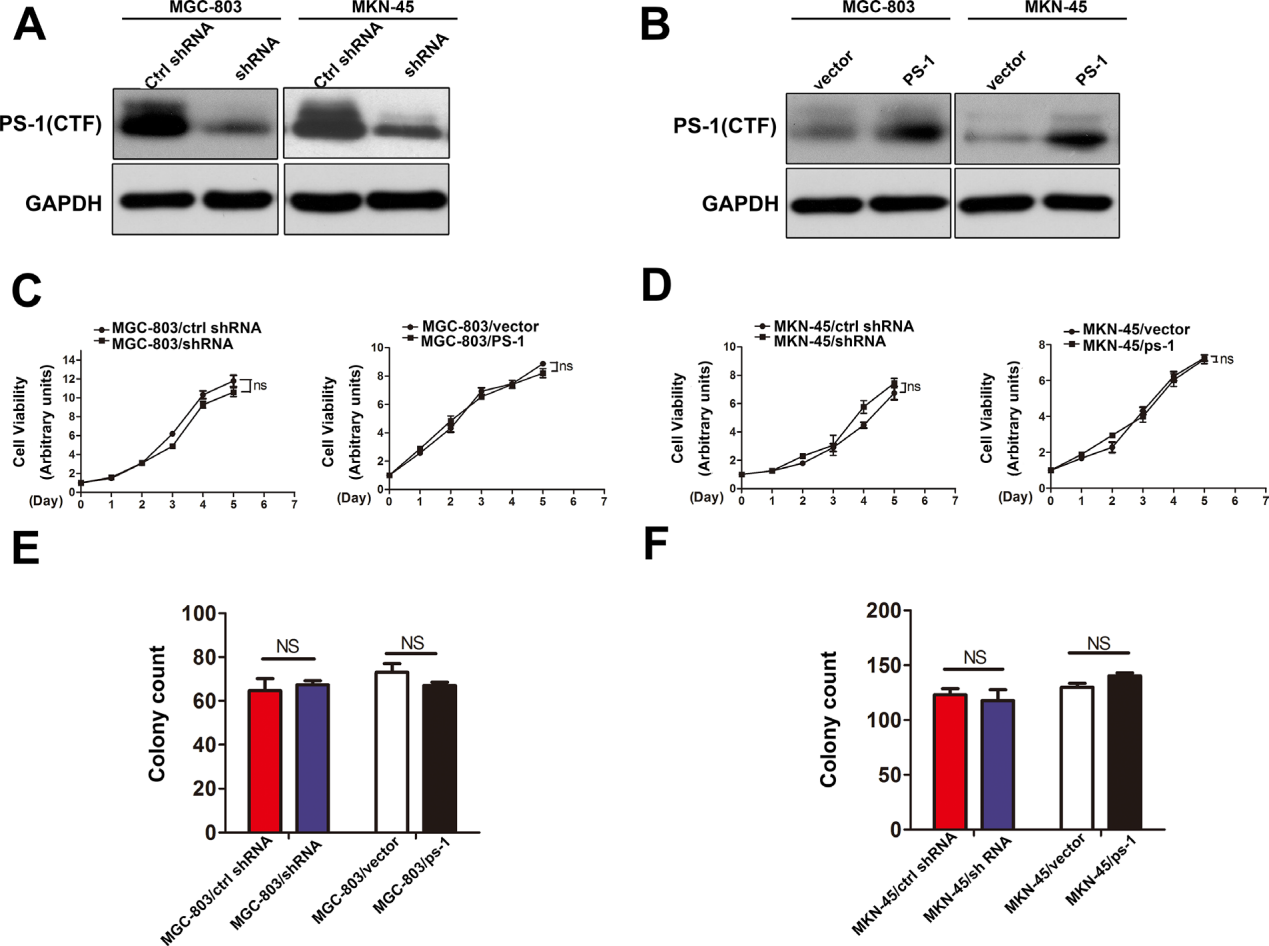
Effects of PS-1 on cell proliferation in human GC cells (**A**) and (**B**) Western blot analysis testing the expression of PS-1(CTF) in MGC-803 and MKN-45 stable cell lines. GAPDH was used as a loading control. (**C**) and (**D**) Effects of PS-1 overexpression and knockdown on cell growth using CCK8 assay (NS: *p* > 0.05). (**E**) and (**F**) Effects of PS-1 knockdown and overexpression on MGC-803 and MKN-45 cell growth using the plate colony formation assay. The same experiment was performed on MKN-45 stable cell lines of overexpression and knockdown PS-1. The data represents mean ± SD of three independent experiments.

Because PS-1 is significantly upregulated in GC, it may act as a proto-oncogene. To determine the function of PS-1 in GC cell lines, we chose two high PS-1 expressing cell lines, MGC-803 and MKN-45, to generate a cell model. After testing the silencing efficacy of 4 pairs of shRNA, we selected the most effective shRNA pair for our application. PS-1 knockdown and PS-1 expressing lentivirus systems were infected into MGC-803 and MKN-45 cells to generate our cellular models (MGC-803/PS-1shRNA, MKN-45/PS-1shRNA, MGC-803/PS-1, and MKN-45/PS-1). The effects of silencing and overexpression were quantified via intensity comparison (Figure 2A and 2B, respectively, *p* < 0.05).

Our clinical data analysis showed no correlation between PS-1 and tumor size. Therefore, we first examined whether PS-1 could affect the viability of GC cells by a CCK8 assay. As shown in Figure 2C, neither knockdown nor overexpression of PS-1 had an effect on MGC-803 cells (*p* > 0.05); the same result was confirmed in MKN-45 cells (Figure 2D, *p* > 0.05). The colony formation assay demonstrated that MGC-803/PS-1 shRNA formed colonies comparable to the control cells. Consistently, PS-1 overexpression also yielded no effect on the colony formation of MGC-803 cells (Figure 2E, *p* > 0.05). Similarly, no difference was observed in MKN-45 cells (Figure 2F, *p* > 0.05). These findings indicate that PS-1 does not affect cell growth in GC cells *in vitro*.

### PS-1 enhances GC cell motility

**Figure 3:**
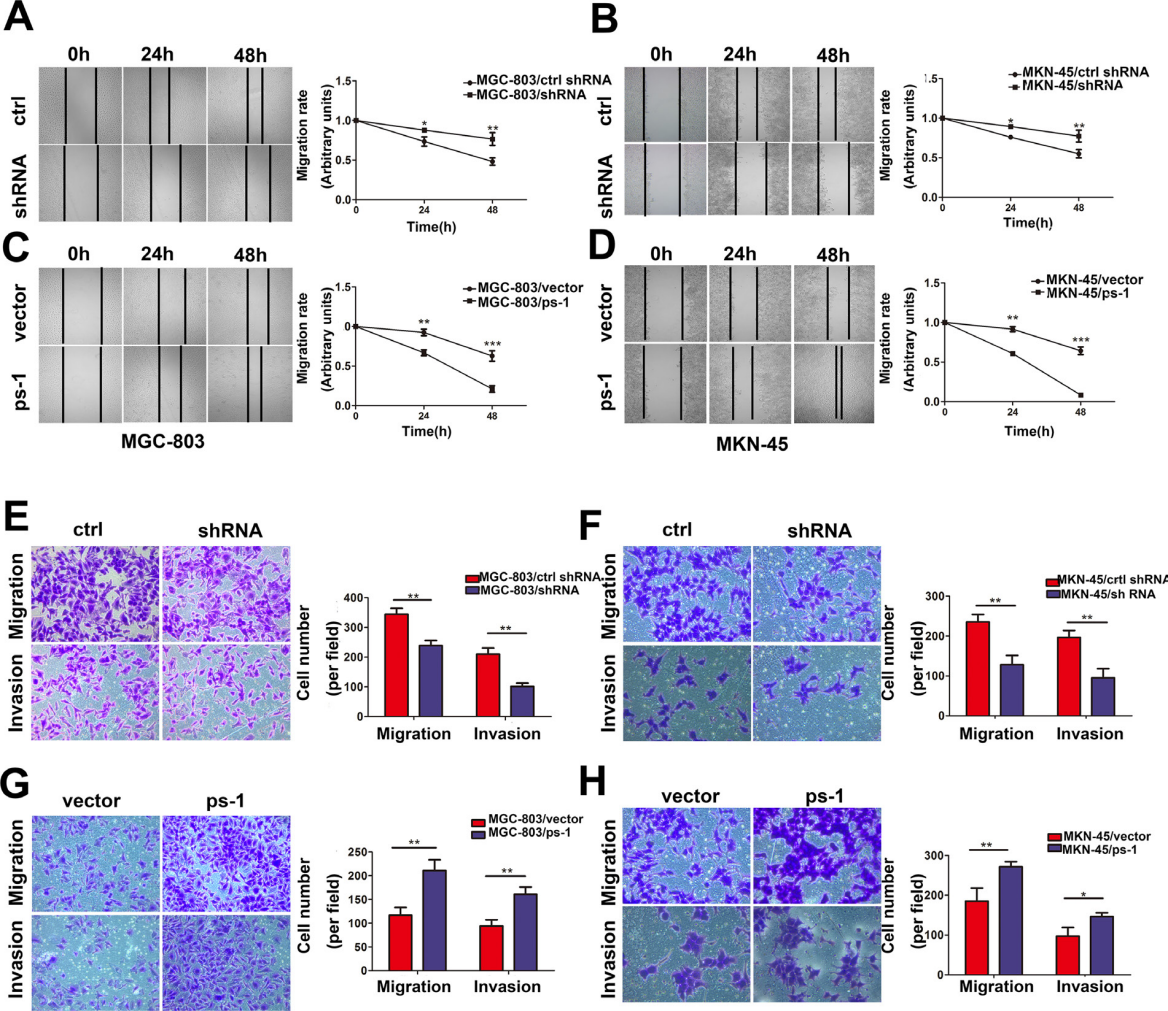
Effects of PS-1 on migration, invasion ability in GC cells (**A**) (**B**) (**C**) and (**D**) Wound healing assay with GC cells. Microscopic observations were recorded 0, 24 and 48 hours after scratching the cell surface (100×). A representative image from every independent experiment is shown (By Left). The distances between wound edges of gastric cancer cells at 0, 24 and 48 hours (MGC-803, left, MKN-45, right). *p < 0.05; **p < 0.01;***p < 0.001. (**E**) Representative images of migrated/invaded MGC-803 cells through chambers' membrane (200×). Histograms showed the numbers of migration cells and invasion cells in the knockdown group (By the left), Cell numbers were counted in five randomly selected microscopic fields (**p < 0.01). (**G**) Representative images of migrated/invaded MGC-803 cells through chambers' membrane. Histograms showed the numbers of migration cells and invasion cells in the overexpression group (By the left), Cell numbers were counted in five randomly selected microscopic fields (**p < 0.01). (**F**) and (**H**) The similar data was obtained in MKN-45 cells. The data is shown as mean ± SD of three independent experiments.

PS-1 expression positively correlated with lymph node metastasis; thus, we further investigated whether PS-1 interfered with the potential motility of GC cells. We performed wound healing assays to verify the migratory speed of the stable cell lines. A marked correlation between the expression of PS-1 and the motility of GC cells was observed. Upregulation of PS-1 dramatically increased migratory capabilities in both MGC-803 and MKN-45 cells compared with the control groups (*p* < 0.01). Similarly, PS-1 downregulation decreased the migratory speeds of both MGC-803 and MKN-45 cells, which were significantly different from those of matched control groups (Figure 3A and 3B, *p* < 0.05). A time-dependent alteration was also observed in our cell models.

To further characterize the effects of PS-1 on cell motility, we implemented a Transwell assay. For MGC-803/ctrl shRNA, the counts of migrating or invading cells were higher than for MGC-803/shRNA (Figure 3E). To confirm this result, the same experiment was performed in the MGC-803/PS-1 group. Upregulation of PS-1 exhibited a higher invasive and migratory capacity than the vector control cells, with a nearly 2-fold increase (Figure 3G). The same phenomenon was observed in the MKN-45 stable cell lines (Figure 3F and 3H). These results suggest that aberrant expression of PS-1 could promote motility in GC cells.

### PS-1 promotes GC metastatic ability in a mouse model

**Figure 4:**
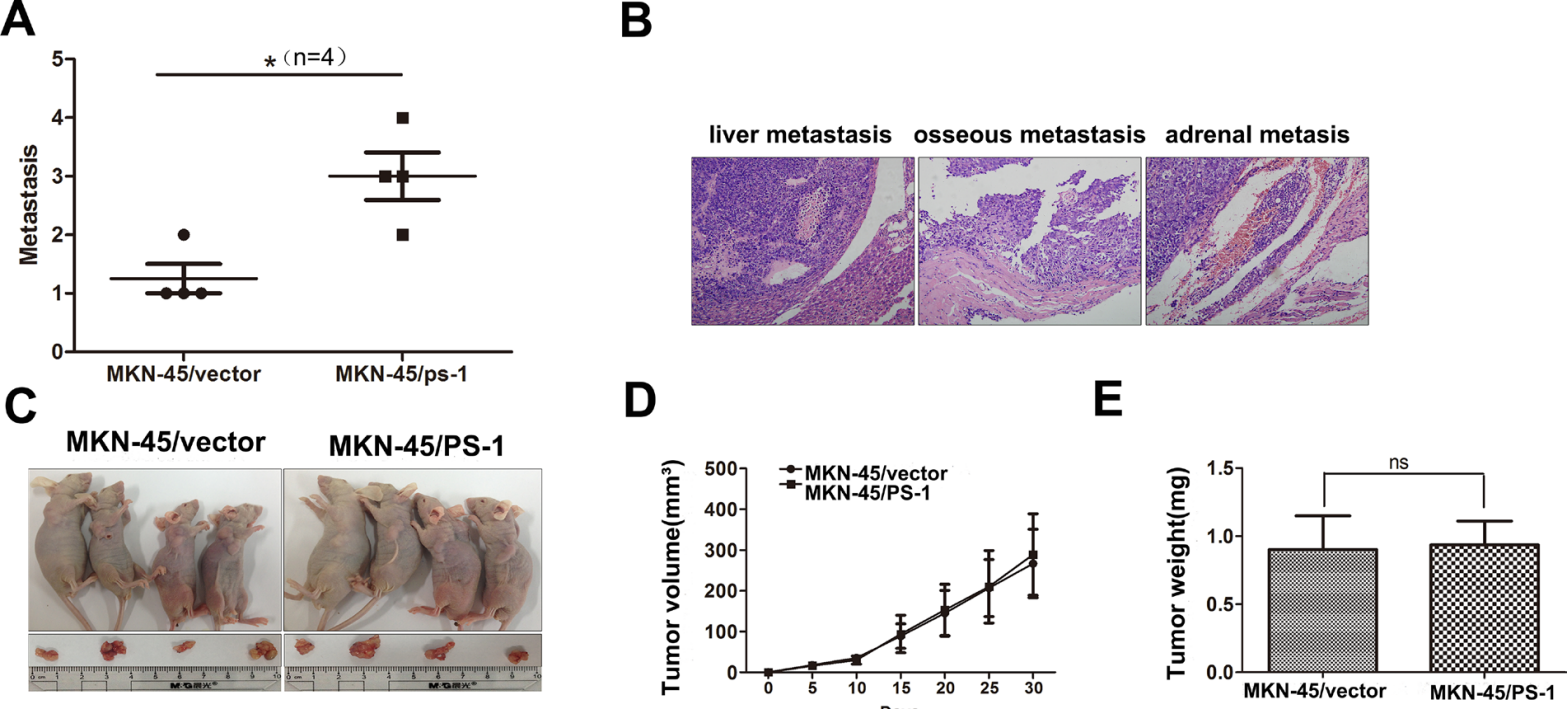
PS-1 enhances the colonization of MKN-45 cells, but not proliferation *in vivo* (**A**) Quantification of the number of macrometastases in the whole body of mice 8 weeks after being injected with MKN-45/PS-1and MKN-45/vector cells via tail vein (*p < 0.05, *n* = 4 per group). (**B**) Upon being euthanized, colonization of tumors were analyzed by histologically. Liver, osseous and adrenal metastasis was presented. (**C**) (**D**) and (**E**) Subcutaneous xenograft model shows no significant difference in tumor growth (*p* > 0.05).

Based on our findings *in vitro*, we investigated the effects of PS-1 on the metastatic colonization of GC cells by intravenously injecting MKN-45/PS-1 cells and MKN-45/vector control cells into the lateral tail veins of nude mice. Eight weeks after injection, the mice were sacrificed to evaluate metastatic nodules throughout the entire body. Mice injected with MKN-45/PS-1 cells harbored more metastatic foci throughout their bodies compared with those injected with the MKN-45/vector control cells (Figure 4A, *p* < 0.05). Metastatic nodules were detected in the liver, bone and adrenal gland and were confirmed by HE staining in the MKN-45/PS-1 group (Figure 4B). Similar results were obtained in the MKN-45/PS-1 shRNA group. We also verified the effect of PS-1 on cell growth *in vivo*. These results demonstrate that tumors grew equally fast between the MKN-45/vector injected mice and the MKN-45/PS-1 injected mice (Figure 4C, 4D and 4E, *p* > 0.05). The same phenomenon was observed in the MKN-45 cells of silencing. (Supplementary Data 3). The *in vivo* data were consistent with our *in vitro* results, further confirming that PS-1 promotes metastasis without affecting cell proliferation in the development of GC.

### PS-1 affects the regulation of E-cadherin cleavage and β-catenin nuclear accumulation in GC

**Figure 5:**
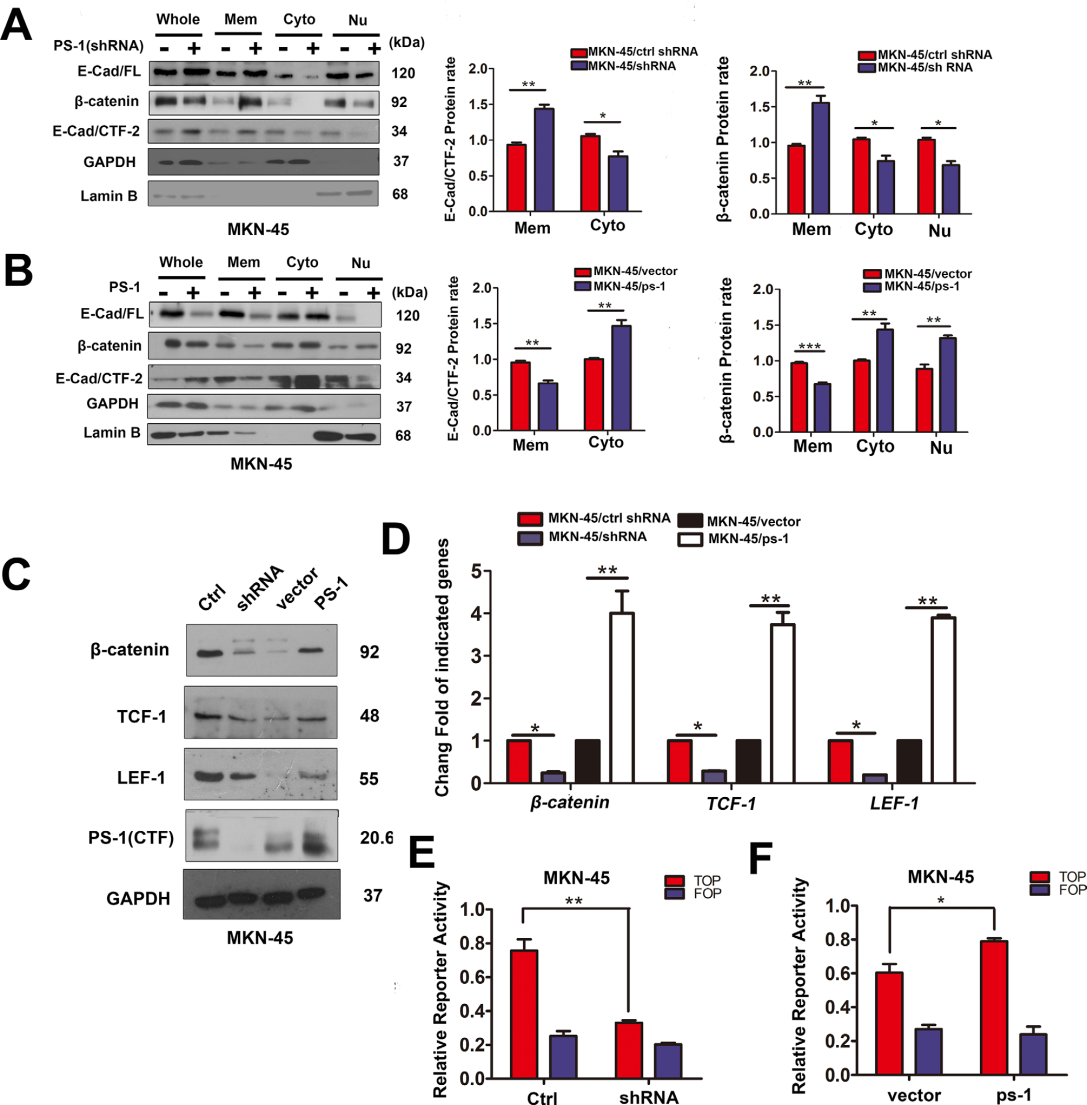
PS-1 affects the regulation and location of E-cadherin and β-catenin in GCs (**A**) and (**B**) Western blot analysis testing the expression of E-cad (cadherin)/FL (full-length), β-catenin, E-cad /CTF-2 (c-terminal fragment-2) and PS-1(CTF) in Mem (membrane), Cyto(cytoplasm) and Nu(nuclear) via Subcellular Fraction Assay. GAPDH as control in cytoplasm and Lamin B in nuclear. (**C**) Western blot analysis of β-catenin, TCF-1 and LEF-1 expression in MKN-45 cells. The quantified analysis of the expression of TCF-1, LEF-1 and β-catenin have been presented on (**D**). (**E**) Knockdown of PS-1 inhibited the TOP-flash reporter in MKN-45 cells. In contrast, overexpress PS-1 activated TOP-flash reporter in MKN-45 cells (**F**). MKN-45/shRNA and MKN-45/PS-1 cells and their control cells were transfected with TOP-flash reporter gene, TK Renilla as a control. *p < 0.05; **p < 0.01;***p < 0.001.

Based on the effect of PS-1 on the invasion and metastasis of GC cells, we investigated whether PS-1 plays roles in dissociating the E-cadherin-β-catenin adhesion complex in GC by performing cytosolic/nuclear fractionation. As shown in Figure 5A, when we silenced PS-1, both E-cadherin (full-length) and β-catenin accumulated on the membrane and decreased in the cytoplasm (*p* < 0.05). Furthermore, E-cad/CTF-2 more strongly localized to the membrane (*p* < 0.01) along with β-catenin (*p* < 0.05). In contrast, overexpression of PS-1 caused the reverse phenomenon (Figure 5B), as additional PS-1 enhanced the accumulation of E-cad/CTF-2 in the cytoplasm (*p* < 0.01) and β-catenin in the nucleus (*p* < 0.01). A luciferase reporter assay showed that the additional β-catenin in the nucleus indeed activated the TOP-flash reporter, whereas the reporter was inhibited when PS-1 was silenced (Figure 5E and 5F, *p* < 0.01 and *p* < 0.05, respectively). A similar result was observed by analyzing TCF-1 and LEF-1 using western blotting (Figure 5C and 5D, *p* < 0.01). These results indicate that PS-1 affects the tumorigenesis of GC by dysregulating the transfer of the E-cadherin binding partner β-catenin to the nucleus and thereby activating the TCF/LEF-1 transcriptional activator, which may promote GC invasion and metastasis.

### PS-1-mediated E-cadherin cleavage is dependent on γ-secretase activity

**Figure 6:**
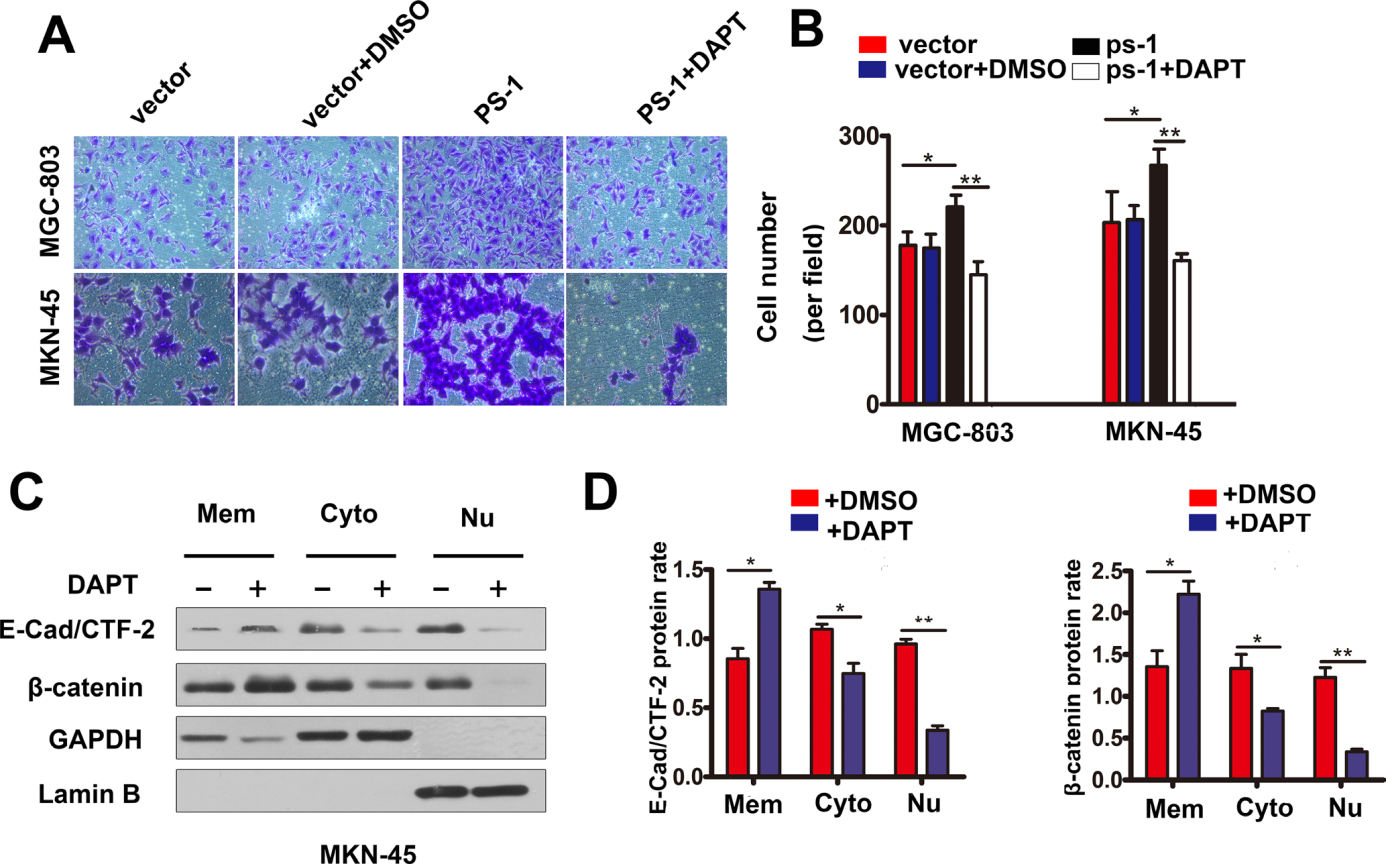
DAPT inhibits gastric cancer cell invasion thought the same way of knockdown PS-1 (**A**) and (**B**) DAPT reversed the invasion ability in MKN-45/PS-1 cells. (**C**) and (**D**) Western blot analysis testing the expression of β-catenin, E-cad /CTF-2 (c-terminal fragment-2) in Mem(membrane), Cyto(cytoplasm) and Nu(nuclear) via Subcellular Fraction Assay when treated with DAPT in MKN-45 at resting stage. GAPDH as control in cytoplasm and Lamin B in nuclear. *p < 0.05, **p < 0.01, ***p < 0.001.

In this study, we used the γ-secretase inhibitor DAPT to confirm that the PS-1-mediated cleavage of E-cadherin depended on the activity of γ-secretase. The results are shown in Figure 6A and 6B; Transwell Matrigel invasion assays demonstrated that DAPT could reverse the invasion capacity of MKN-45 cells when PS-1 was overexpressed (*p* < 0.01). Furthermore, the amounts of E-cad/CTF-2 and β-catenin were decreased when MKN-45 cells were treated with DAPT in combination with PS-1 silencing (Figure 6C and 6D, *p* < 0.05 and *p* < 0.01, respectively). These results indicate that the cleavage function of PS-1 depends on γ-secretase activity in GC.

## DISCUSSION

In this study, we investigated the role of PS-1 in the carcinogenesis of GC. The potential mechanisms underlying the transformation of GC cells, with respect to invasion and migration capabilities, are under preliminary research.

Although several studies have reported a function for GSIs in GC treatment, they have been exclusive to investigating Notch signaling rather than the dysregulation of PS-1, the core subunit of γ-secretase, in the tumorigenesis of GC [23, 25, 26]. Based on our large number of fresh GC samples and multiple methods of investigation, we determined that PS-1 is dramatically upregulated in GC tissues and cell lines compared with their benign counterparts. Combined with clinical parameters and survival results, it is clear that the PS-1 expression level is closely correlated with lymph node metastasis. Higher expression of PS-1 in patients was correlated with a lower survival rate, which could be attributed to ubiquitous lymph node metastases being a negative regulator of life span. Thus, PS-1 is recognized as a therapeutic target and proto-oncogene in GC, consistent with previous studies [13, 20, 27].

Based on previous clinical research results, we performed a tumor migration assay. By constructing stable cell lines, we were able to establish a strong relationship between the expression of PS-1 and an altered capacity for cell migration and invasion, both *in vitro* and *in vivo*. PS-1 overexpression in GC cells potentiated migration rates and depth of invasion. By contrast, the opposite results were observed when PS-1 was silenced in GC cells. In our mouse model, PS-1 overexpression in GC cells injected into the tail vein showed diffuse infiltration from the liver to the abdominal cavity and kidney, whereas no solid tumor formed when PS-1-silenced GC cells were injected, compared with the control groups. The PS-1/γ-secretase complex has more than 90 identified substrates, most of which are involved in tumorigenesis [28]. Notably, the classical cadherins are primarily cleaved by the PS-1/γ-secretase complex, which induces the alteration of cell-cell adhesion. This superfamily is classified as a single-pass transmembrane glycoprotein with two portions: the extracellular domain that mediates intercellular adhesion and the intracellular domain that contains the binding sites for β-catenin and p120-catenin, which are linked to the actin cytoskeleton [29]. The PS-1/γ-secretase complex contributes to P-cadherin, N-cadherin and E-cadherin processing and subsequently regulates cell motility and invasive ability [30–32]. Previous studies have reported that PS-1 has conflicting roles in the tumorigenesis of different cancers. These conflicting roles could be attributed to micro-environment and tissue specificity. Depending on the status of cell-cell adhesion, PS-1 has two distinct activities: (1) under conditions that favor cell-cell disassociation, PS-1 primarily disassembles cadherin-based adherens junctions (CAJs), which triggers the cleavage of membrane-associated, full-length E-cadherin and increases free cytosolic β-catenin levels, thus facilitating cell migration and invasion; and (2) under conditions that contribute to cell-cell adhesion, PS-1 forms a complex with CAJs and stabilizes this complex [8, 33–35].

In this work, we observed a negative correlation between the expression of PS-1 and full-length E-cadherin. Conversely, a positive correlation was observed between PS-1 and β-catenin. Furthermore, PS-1 enhanced the cytosolic and nuclear accumulation of β-catenin and boosted the expression of E-cad/CTF-2 in the cytoplasm. Most importantly, the nuclear accumulation of β-catenin activated T-cell factor/lymphocyte enhancer factor-1 (TCF-1/LEF-1), which acts as a transcriptional activator. We hypothesize that the potential mechanism by which dysregulation of PS-1, E-cadherin and β-catenin alters GC cell motility and metastasis may be related to these factors: (1) the cleavage of membrane-associated, full-length E-cadherin is accompanied by loss of cell-cell adhesion and disintegration of CAJ; and (2) the cytosolic and nuclear abundance of free β-catenin enhances tumorigenesis via the activation of the TCF/LEF-1 transcriptional activator [14, 36, 37]. Consequently, we show that, with increased soluble β-catenin levels in the cytoplasm, APC, GSK3β and p-GSK3β were up-regulated (Supplementary Data 4), thereby leading to the degradation of β-catenin [38]. To explain the upregulation of APC, GSK3β and p-GSK3β, we hypothesize that there may be a negative feedback loop between soluble β-catenin and the APC/GSK3β complex.

DAPT, a type of GSI, has been studied in our system. In contrast to previous studies, DAPT inhibits the tumorigenesis of GC cells through alterations in Notch signaling or EMT [15, 22]. We observed that membrane-associated E-cadherin increased after DAPT treatment and that GC cell motility and metastasis were also altered, further supporting our hypothesis and confirming that the effects of PS-1 depend on γ-secretase.

Notably, PS-1 deficient mice present severe developmental defects and perinatal lethality due to impairments in neurogenesis and skeleton formation [39]. In skin tumorigenesis, PS-1 can cause β-catenin /LEF mediated cyclin D1 activation, thus arresting cell entry from G1 into S phase and regulating cell proliferation [15]. Here we demonstrate that PS-1 does not affect cell viability in the development of GC. No correlation was observed between PS-1 expression and tumor size from the clinical data analysis. The same conclusion was also drawn from our *in vivo* and *vitro* experiments, and no differences were observed between our cells and mouse models (Supplementary Data 3).

In conclusion, we demonstrated that PS-1 enhances GC cell invasion and migration without altering cell proliferation, both *in vitro* and *vivo* following either up- or down-regulation. We propose that PS-1 is associated with CAJ disassembly and that its enzymatic role contributes to the relocalization of β-catenin from the cell membrane to the nucleus and drives cancer progression by triggering TCF/LEF-1 activation. DAPT suppresses the tumorigenesis of GC cells, in part because of increased membrane-associated, full-length E-cadherin levels. These results indicate a potential for the suppression of PS-1 in therapeutic applications for GC.

## MATERIALS AND METHODS

### Clinical tumor tissues

All the GC samples were from FuJian Medical University Union Hospital, China. Among them, the 204 paraffin-embedded GC tissues were collected from October 2006 to September 2007. And 136 fresh GC tissues were recruited randomly between June 2010 and June 2013. The GC tissue samples from those patients were confirmed by pathological diagnosis. The corresponding non-tumor samples were located at least 5 cm from the gastric tumor. All of the specimens, including tumor and paired non-tumor tissues, were stored in liquid nitrogen after resection until protein or RNA extraction. None of the patients in our study received preoperative chemotherapy, radiotherapy, or other biological treatment. All the patients underwent standard D2 lymph node dissection with curative resection (R0). Postoperative adjuvant chemotherapy was performed with 5-fluorouracil-based drugs plus Oxaliplatin in advanced cases. The study was approved by the Fujian Medical University Union Hospital institutional review board, and written informed consent was obtained from all participants. The clinical and pathological staging were performed according to the American Joint Committee on Cancer (AJCC) seventh edition of GC TNM Staging [40].

### Patient follow-up method

All patients were systematically followed up by trained doctors based on institutional follow-up protocol, in several ways via outpatient service, letter, telephone, mail or visiting. Until June 2015, all of the surviving patients were followed up for more than 5 years. Among the 204 patients, 201 (98.5%) were followed up, and 3 (1.5%) were lost to follow-up.

### Immunohistochemical analysis

Immunohistochemical staining for PS-1 was performed on formalin-fixed, paraffin-embedded gastric tissue sections (3 μm thick, tumor or normal). Paraffin-embedded tissue sections from GC specimens were given a heat pretreatment of 70°C for one hour, then dewaxed in xylene, rehydrated in an ethanol series (100–50%) and treated in 0.01 mol/L citrate buffer (pH6.0) for antigen retrieval. After inhibition of endogenous peroxidase activity for 30 min with methanol containing 0.3% H2O2, the sections were stained with a rabbit anti-PS-1 monoclonal antibody (1:200, Abcam, ab76083) at 4°C overnight. The following experimental procedure was on the basis of the manufacturer's instructions of the LSAB+ kit (Dako, USA). The PS-1 protein expression was immunohistochemically demonstrated as yellowish to brown staining in the cytoplasm and membrane of gastric glandular cells. By Two pathologists, blinded to the clinical data, reviewed the immunoreactivity for PS-1 protein under a light microscope, and the protein expression was scored independently according to the intensity of cellular staining and the proportion of stained tumor cells. The staining intensity was scored as 0 (no staining), 1 (weak staining, light yellow), 2 (moderate staining, yellow brown), and 3 (strong staining, brown), and the proportion of stained tumor cells was classified as 0 (≤ 5% positive cells), 1 (6% to 25% positive cells), 2 (26% to 50% positive cells), and 3(≥ 51% positive cells). The product of the scores for intensity and proportion was used to signify the level of protein expression. A score of 3 or less was considered low PS-1 expression, and a score of 4 or more was considered high PS-1 expression.

### Western blot analysis

Fresh tissues and cells were homogenized in RIPA protein lysis buffer containing protease inhibitors at 4°C for 30 min before centrifugation at 12,000 g for 10 min at 4°C. The supernatants, representing whole-cell lysates, were prepared for use in subsequent experiments. The protein concentration was measured using the BCA Protein Assay Kit (Thermo). A total of 40 μg protein from each sample was denatured and loaded into each well, separated by SDS-PAGE, and transferred to a polyvinylidene difluoride membrane (Millipore, Billerica, MA). Subsequently, the membrane was blocked with 5% nonfat milk at room temperature for 1 hour. The membrane was incubated with rabbit anti-PS-1 (1:1,000; Abcam, ab76083) or rabbit anti-GAPDH (1:1,000; Abcam, ab181602) primary antibodies overnight at 4°C. After washing with wash buffer (10 mmol/L Tris-HCl, 150 mmol/L NaCl, and 0.1% Tween 20), the membrane was further incubated with horseradish peroxidase-conjugated goat anti-rabbit IgG (Sigma, St. Louis, MO) at a 1:2,000 dilution for 1 hour at room temperature. Subsequently, the membrane was washed for 30 min with wash buffer and detected using enhanced chemiluminescence (Amersham Corporation, Arlington Heights, IL, USA). Antibodies from Cell Signaling Technology (USA) were also used for specific molecules like: APC(cat 13329), GSK-3β (cat 5676), P-GSK-3β (Ser9, cat 9322), TCF-1(cat 2203), LEF-1 (cat 2286).

### Cell lines

All the six gastric cancer cell lines were purchased from Shanghai Institute of Cell Biology, China. Wherein, BGC-823 (poorly differentiated cell lines), SGC-7901 (moderately differentiated cell lines) and MGC-803 (signet cell lines) were established in China and widely used in the study of gastric cancer [41]. MKN-45 (poorly differentiated cell lines) derived from liver metastatic masses, and MKN-28 (moderately differentiated cell lines) from lymph node metastatic masses, both of them were established in Japan [42]. Cell lines were cultured in DMEM (Gibco) medium supplemented with 10% FBS (Gibco) and maintained in a humidified atmosphere containing 5% CO_2_ at 37°C.

### Plasmid construction and transfection

Lentivirus expressing PS-1 shRNA or control shRNA were purchased from GeneChem Corporation (Shanghai, China, GCPL45123). The PS-1 cDNA ORF was cloned into the pHBLV-IRES-ZsGreen-PGK-Puro plasmid (Hanbio™) for lentiviral production. Stable cell lines were screened with puromycin and identified by western blotting.

### Membrane/cytosolic/nuclear fractionation assay

The assays were performed according to the manufacturer's instructions for the Qproteome Cell Compartment Kit (Qiagen). A total of 40 μg protein from each fraction was denatured and loaded into each well, and SDS-PAGE and western blotting were conducted as described above. Antibodies against E-cadherin (full-length and CTF-2) were purchased from BD Biosciences, anti-β-catenin was purchased from abcam (ab32572), and anti-lamin B was purchased from Santa Cruz Biotechnology.

### Cell proliferation assay

Cells were seeded onto 96-well plates at a final density of 1.0 × 10^3^ viable cells/well and incubated for 5 days. Cell proliferation was then measured by using the cell counting kit CCK-8 (Donjindo, Kumamoto, Japan). The absorbance(A) at a wavelength of 450 nm was measured using a microplate reader (Bio-Tek, Winooski, VT, USA).

### Colony-formation assay

For the colony-formation assay, cells were resuspended in DMEM containing 10% FBS and placed into 6-well plates at 1 × 10^3^ cells/well. The cells were incubated for 2 weeks and then stained with crystal violet. Colonies containing 50 or more cells were counted.

### Wound-healing assay

Cells were seeded into 6-well plates and cultured until they reached confluence. Wounds were scratched onto the monolayer of cells using 20-μL pipette tips. The plates were washed twice with fresh medium to remove non-adherent cells after the cells had been cultured for 0, 24 or 48 hours, and the plates were then photographed

### Cell migration and invasion assay

Cell migration and invasion was measured using a transwell chamber (8 μm, 24-well format; Corning, Lowell, MA, USA). To measure migration, 8 × 10^4^ cells were resuspended in 0.3 mL of serum-free medium and added to the upper chamber, while 0.8 mL of medium containing 10% FBS was added to the lower chamber. Cells were incubated for 24 hours. To measure invasion, diluted Matrigel (BD Biosciences) was used to coat the insert chamber membrane. Cells were cultured for 48 hours under the same conditions. For the pharmaceutical experiment with DAPT (SelleckChem, Houston, TX, USA), cells were incubated with DAPT(30 uM) for 72 hours before proceeding with the transwell assay described above, in which the lower and upper chambers both contained DAPT. Finally, cells that migrated or invaded into the lower chamber were fixed with methanol, stained with crystal violet and counted in 5 random fields.

### Luciferase reporter assay

Cells were plated at a subconfluent density and co-transfected with 0.5 μg of the reporter plasmid, 0.5 μg of expression vector, and 0.5 μg of the Renilla luciferase-encoding plasmid pRL-TK (as an internal control for transfection efficiency). Cell lysates were prepared 24 hours after transfection, and the reporter activity was measured using the Dual-Luciferase Reporter Assay System (Promega, Madison, WI). Transfections were performed in triplicate and were repeated 3 times to ensure reproducibility.

### *In vivo* tumorigenesis

SPF-grade male BALB/c nude mice were purchased from the Institute of Zoology, Chinese Academy of Sciences. Cells (3 × 10^6^) were resuspended in 0.2 ml of DMEM and were subcutaneously injected into mice. The length (L) and width (W) of each tumor were measured every 5 days with calipers, and the volume was calculated using the formula: (W + L) / 2 × W × L × 0.5236. For the metastasis experiment *in vivo*, 5 × 10^6^ stable cells (including cells subjected to knockdown or overexpression) were resuspended in 0.3 mL of DMEM and injected intravenously into 4-week-old male nude mice (*n* = 4 per group). The mice were anesthetized after 8 weeks, and metastatic nodules were counted throughout the entire body.

### Statistical analysis

All measurement data are presented as the means ± SE and were analyzed using SPSS 17.0 for Windows (SPSS, Chicago, IL) and Prism 5.0 software (GraphPad). The relationships between the PS-1 expression level and clinicopathologic parameters were calculated with the Pearson *χ2* test. Survival curves were explored by the Kaplan–Meier method, and differences between 2 groups were evaluated by the log-rank test. Comparisons were performed using Student's *t* test (2 groups) or one-way ANOVA (multiple groups). The statistical significance threshold was set as *p* < 0.05.

## SUPPLEMENTARY MATERIALS DATAS


