# Supplementary material for: The expression of presenilin 1 enhances carcinogenesis and metastasis in gastric cancer
Source: Oncotarget. 2016 Feb 10;7(9):10650–62. doi: 10.18632/oncotarget.7298 (PMC4891148; doi:10.18632/oncotarget.7298)
Supplement: Supplementary file 1 [file oncotarget-07-10650-s001.pdf]

The expression of presenilin 1 enhances carcinogenesis and metastasis in gastric cancer

Supplementary Materials

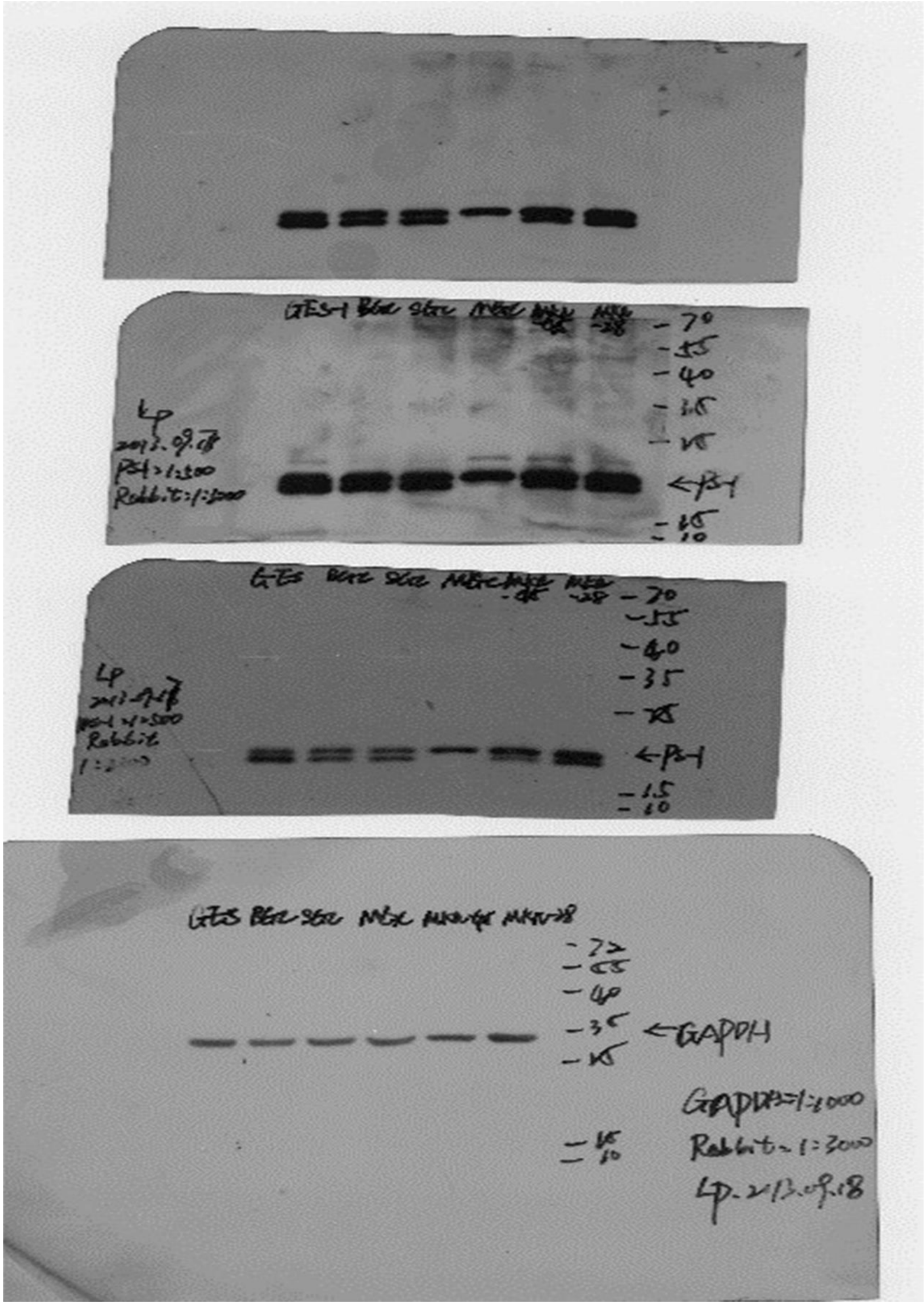

Supplementary Data 1: The photographic plate of the expression level of PS-1(CTF) in different gastric cancer cell lines.

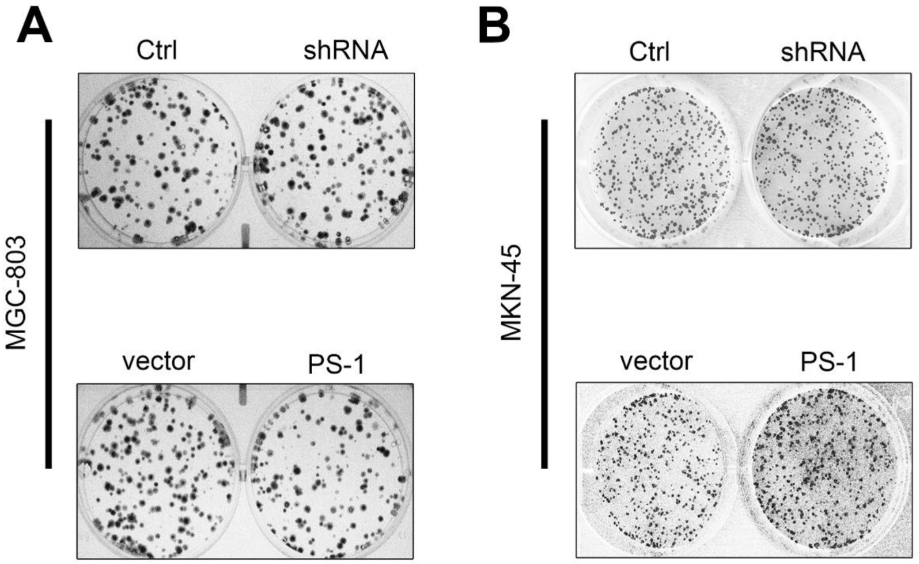

Supplementary Data 2: Effects of PS-1 on cell proliferation in human GC cells. (**A**) and (**B**) The plate colony formation assay showed no significant difference of knockdown and overexpression PS-1.

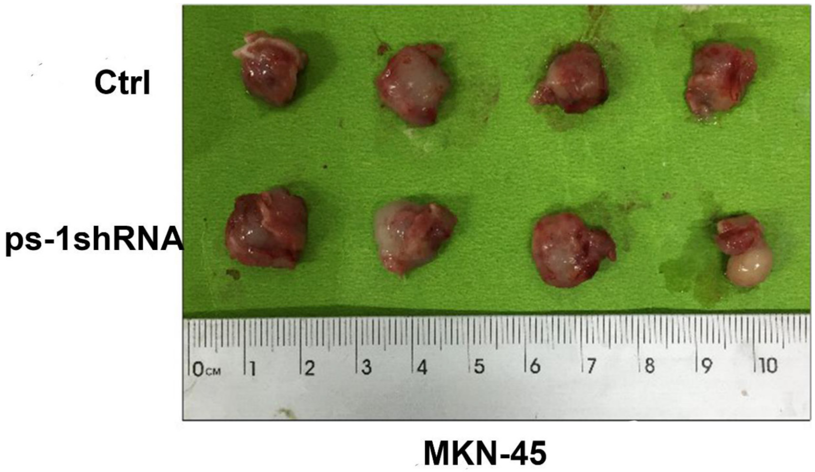

Supplementary Data 3: Xenograft model shows no significant difference of silencing PS-1 in tumor growth (*p* > 0.05).

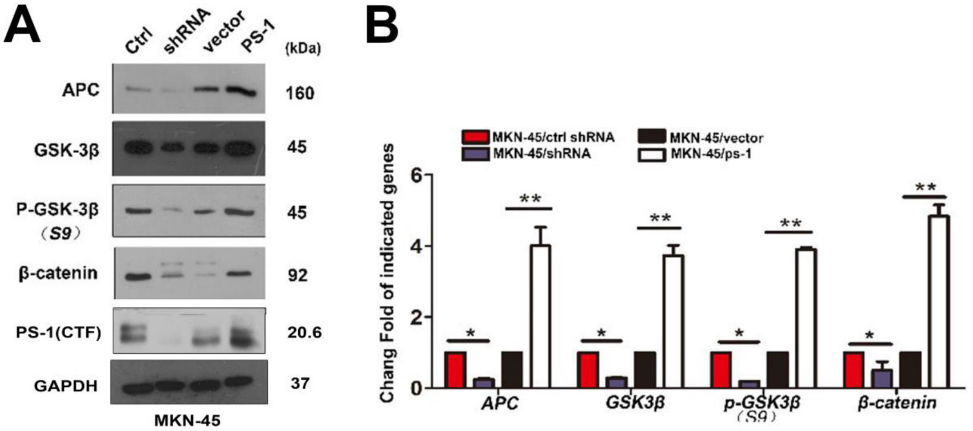

Supplementary Data 4: Western blot analysis of APC, GSK3β, phospho-GSK3β (S9), β-catenin in MKN-45. Under silence and overexpression of PS-1, the expression levels of APC, GSK3β, phospho-GSK3β (S9) and β-catenin were detected via western-blot in (**A**), the quantification of these proteins was analyzed via densitometry shown in (**B**).
